# Identification of candidate biomarkers for NAFLD through bioinformatics analysis and machine learning based on circulating insulin degradation-associated genes

**DOI:** 10.3389/fendo.2026.1774997

**Published:** 2026-05-12

**Authors:** Mingjie Guo, Wei Lou, Xin Song, Dongxin Gao, Guoan Wang, Hanyu Ma, Wenlei Wang, Yongliang Wang

**Affiliations:** School of Basic Medical Sciences, Huaihe Hospital (Zhongzhou Laboratory for Integrative Biology), Henan University, Kaifeng, Henan, China

**Keywords:** bioinformatic analysis, biomarkers, circulating insulin degradation, machine learning, non-alcoholic fatty liver disease, WGCNA

## Abstract

Non-alcoholic fatty liver disease (NAFLD) has become as a metabolic disorder posing a significant threat to public health, with no presently available effective treatment. Circulating insulin degradation constitutes a pivotal process regulating insulin concentration and biological activity in the bloodstream, and its capacity is closely associated with hyperinsulinaemia and hepatic lipid accumulation. Hepatic lipid accumulation represents a key pathophysiological mechanism in NAFLD. Therefore, targeting the circulating insulin degradation pathway may represent a significant therapeutic opportunity for NAFLD. This study employed a multi-omics strategy, incorporating pertinent datasets from the Gene Expression Omnibus (GEO) collection, to investigate the function of circulating insulin degradation in NAFLD. We employed systems biology informatics approaches, including weighted gene co-expression network analysis (WGCNA) and machine learning models, to identify four hub biomarkers: MYO7A, AGTR1, IL1RN, and IGFBP2. We applied Shapley Additive Explanations (SHAP) to interpret the contribution of each gene to the machine learning model. The expression patterns and potential relevance of these hub genes were further assessed in external datasets, cellular models, and animal models. Overall, this hypothesis-generating study identified four candidate genes potentially associated with NAFLD and provided additional insights into the molecular mechanisms underlying disease progression.

## 1 Introduction

The terms metabolic dysfunction-associated fatty liver disease (MAFLD, suggested in 2020) and non-alcoholic fatty liver disease (NAFLD) were replaced by metabolic dysfunction-associated steatotic liver disease (MASLD) in 2023 (1–3). This disease represents a metabolic disorder arising from multifactorial interactions, whose typical pathology is characterized by excessive fat accumulation within hepatocytes, requiring exclusion of liver injury caused by alcohol, specific toxins, or medications (4, 5). Most GEO databases previously employed NAFLD terminology; to maintain consistency, we have retained NAFLD. NAFLD is one of the most prevalent chronic liver diseases, and its prevalence is rapidly increasing worldwide (6). NAFLD is not only a significant precipitating factor for liver failure but is also closely associated with elevated risks of cardiovascular disease, type 2 diabetes, hepatocellular carcinoma, and extrahepatic malignancies. It has emerged as a metabolic disorder posing a grave threat to public health (7, 8).

The maintenance of circulating insulin homeostasis depends on the dynamic equilibrium between its synthesis, secretion, degradation, and clearance, with insulin degradation being a critical component regulating insulin concentration and biological activity in the circulatory system (9). Recently, studies have revealed that impaired insulin degradation correlates with hyperinsulinemia and hepatic lipid accumulation. Liver fat accumulation is a critical pathophysiological mechanism in NAFLD, suggesting that abnormal circulating insulin degradation may contribute to metabolic disease pathogenesis by regulating insulin homeostasis (9–12). Furthermore, a growing body of research in recent years has demonstrated the critical role that circulating insulin degradation plays in hepatic metabolic disorders, suggesting that controlling circulating insulin degradation may be a major prospective treatment target for NAFLD (13). Through bioinformatic analyses of gene expression, co-expression networks, and machine learning models, this study provides insights into potential molecular mechanisms and identifies candidate genes of possible therapeutic relevance, thereby offering a basis for future studies in biomarker discovery and precision medicine.

Using the GEO database, this work methodically examined differentially expressed genes (DEGs) between NAFLD patients and healthy controls. Through the Genecards database, we obtained gene sets associated with circulating insulin degradation. We found crucial module genes using weighted gene co-expression network analysis (WGCNA). We intersected the aforementioned three gene sets to obtain the intersecting genes. We then developed a machine learning model to identify hub genes and verified its effectiveness. We applied Shapley Additive Explanations (SHAP) to interpret each gene’s contribution to the machine learning model. We further validated the hub genes at the dataset, cellular, and animal model levels to ascertain their accuracy.

## 2 Materials and methods

### 2.1 Data collection and processing

**Figure 1:**
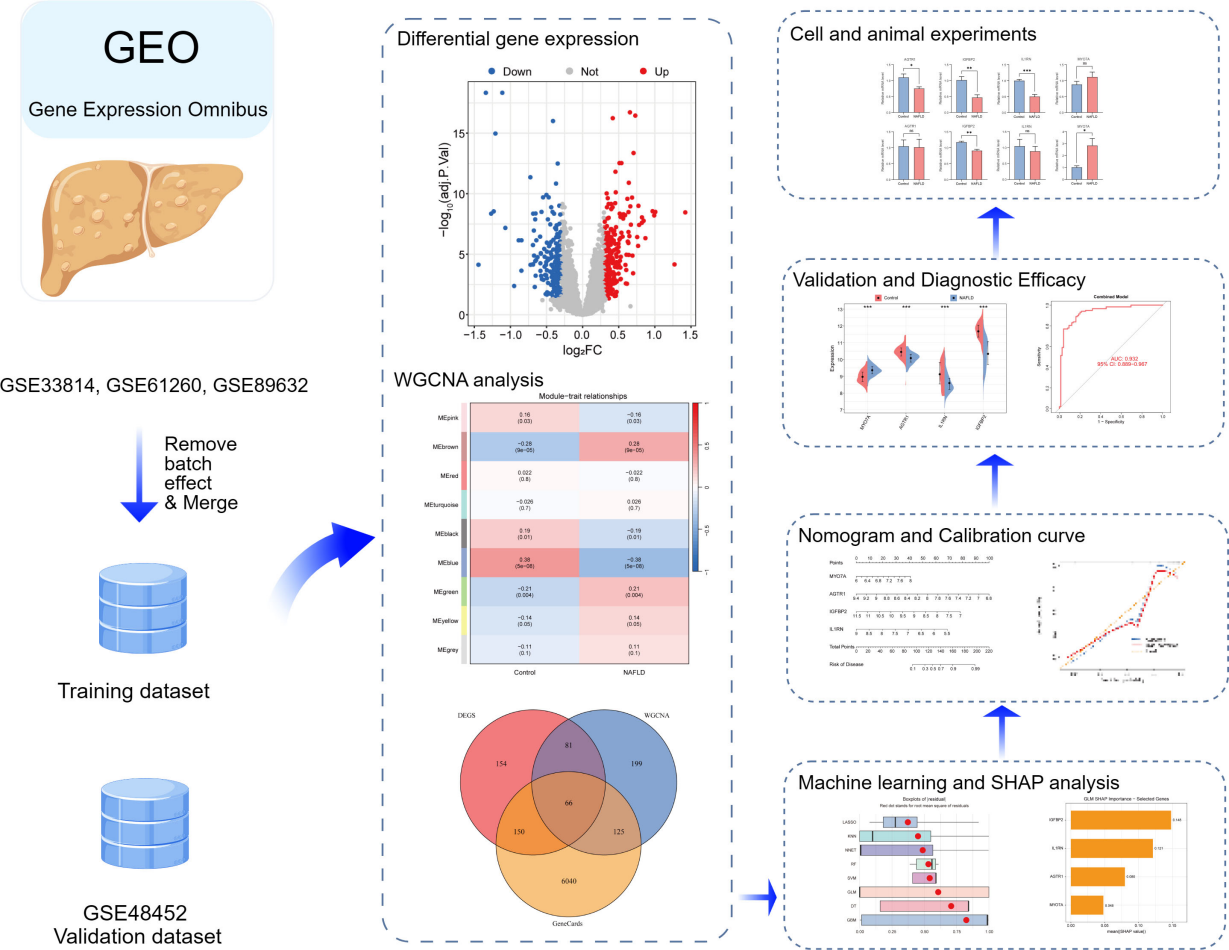
Flowchart for research.

The workflow diagram for this study is shown in **Figure 1**. The data and details were retrieved from the GEO database for NAFLD and control group samples. We obtained the following datasets: GSE33814, GSE61260, GSE89632, and GSE48452. In detail, GSE33814 (GPL6884 platform) comprised 13 healthy controls and 31 NAFLD samples. GSE61260 (GPL11532 platform) included 47 NAFLD cases and 38 healthy controls. GSE89632 (GPL14951 platform) comprised 39 NAFLD and 24 healthy control samples. The combined GSE33814, GSE61260, and GSE89632 datasets formed the training cohort, totaling 75 healthy controls and 117 NAFLD samples. GSE48452 (platform GPL11532) served as the validation cohort, comprising 14 healthy controls and 32 NAFLD samples. Detailed dataset information is presented in **Table 1**. For data preprocessing, probe IDs were mapped to gene symbols using the respective platform annotation files. To ensure a unique expression value for each gene, the avereps function in the “limma” R package was utilized to average the expression levels of multiple probes or duplicate gene entries. Only genes consistently expressed across all training datasets were retained for subsequent integration. To eliminate technical variation, batch effects were corrected using the ComBat function within the “sva” R package. A design matrix including the biological group information (NAFLD vs. Control) was incorporated as a covariate to safeguard meaningful biological signals during the normalization process. DEGs in the combined dataset from the NAFLD and control groups were analyzed using the “Limma” program. The R package “ggplot2” was used to create volcano plots of DEGs (14, 15).

**Table 1:** Basic information of the datasets.

Datasets	Platform	Organism	Control	NAFLD
GSE33814	GPL6884	Homo sapiens	13	31
GSE61260	GPL11532	Homo sapiens	38	47
GSE89632	GPL14951	Homo sapiens	24	39
GSE48452	GPL11532	Homo sapiens	14	32

The Genecards database (https://www.genecards.org/) was used to extract and download genes linked to circulating insulin degradation, choosing those with a correlation score higher than 2. This process yielded 6,381 genes implicated in circulating insulin degradation.

### 2.2 Implementation of WGCNA and identification of key module genes

Finding co-expression gene modules, investigating possible connections between gene networks and important phenotypes, and clarifying the functions of important regulatory genes within these networks are the main goals of WGCNA, a widely accepted systems biology tool. By effectively screening for highly correlated gene clusters, WGCNA reveals the functional synergy exhibited by these gene populations during biological processes. It is noteworthy that WGCNA constructs a weighted gene co-expression network, wherein the connections between genes are not merely simple binary associations. Through the construction of a weighted network, interactions between genes are characterized not only by their presence or absence but also quantitatively reflect the strength of correlation between genes. The R package “WGCNA” was used in our work to conduct consensus clustering analysis (16). Samples were first clustered based on gene expression profiles to eliminate outliers (17). Using the pickSoftThreshold function, which approximates a scale-free network distribution, the optimal power-law exponent was found (18). The “blockwiseModules” function was used to build the scale-free network, and module partitioning analysis was then performed. Topological overlap was used to identify gene co-expression modules (19). The expression profiles of each module were represented by module signature genes, and each module was given a distinct color identifier. Modules exhibiting the highest correlation were designated as key modules.

Subsequently, the 451 DEGs, 6,381 genes associated with circulating insulin degradation, and 471 key module genes were intersected, producing 66 intersecting genes. The CIBERSORT algorithm was utilized to estimate the relative proportions of immune cell subtypes. The LM22 signature matrix, which defines 22 distinct human immune cell subsets, was utilized as the reference for deconvolution. To ensure the reliability of the estimation, only samples with a CIBERSORT output of *P* < 0.05 were included in the subsequent analysis.

### 2.3 Application of machine learning for screening hub genes

Eight machine learning model—Random Forest (RF), Support Vector Machine (SVM), Generalised Linear Model (GLM), Gradient Boosting Machine (GBM), K-Nearest Neighbours (KNN), Neural Network (NNET), Least Absolute Shrinkage and Selection Operator (LASSO), and Decision Tree (DT)—were used in this study (20, 21).

To ensure the robustness of our findings, a nested cross-validation (Nested-CV) framework was employed. The inner loop was dedicated to hyper-parameter tuning and feature selection, while the outer loop provided an unbiased estimation of model stability. Model interpretability was assessed via the “DALEX” package, utilizing residual distribution analyses and root mean square loss to determine feature importance. After identifying the optimal model, its generalizability was rigorously tested on an independent external validation set (GSE48452). The discriminative power was quantified by the Area Under the Curve (AUC). Furthermore, the clinical utility of the final diagnostic signature was evaluated through calibration curves to assess the agreement between predicted and observed risks, and Decision Curve Analysis (DCA) to determine the clinical net benefit. Finally, a predictive nomogram was constructed based on the identified four-gene signature (MYO7A, AGTR1, IL1RN, and IGFBP2) to facilitate clinical decision-making (22).

### 2.4 ROC curve analysis

The ROC curve serves as an established graphical analytical tool for assessing the predictive efficacy of hub genes. In order to fully represent the predictive ability of hub genes under all possible classification thresholds, its fundamental idea is to plot the connection between true positive rate (TPR) and false positive rate (FPR) across various classification thresholds. The “pROC” package was used to create the ROC curve for this study (23).

### 2.5 The interpretability of optimal machine learning models

SHAP breaks down an individual sample’s prediction into the additive contributions of each input feature. It is based on the Shapley value from cooperative game theory. This provides a consistent, comparable attribution value (SHAP value) for each feature, where positive and negative values respectively indicate that the feature increases or decreases the predicted risk. At the global level, the average absolute SHAP value for each feature is calculated to assess overall importance and rank features, revealing the overall association between feature values and model outputs, including potential non-linear relationships. At the local level, SHAP values for each feature are computed and visualized for individual samples, explaining why that particular sample received a specific predicted outcome (24).

### 2.6 Functional enrichment analysis using Kyoto Encyclopedia of Genes and Genomes and Gene Ontology

In order to assess gene-associated biological processes (BP), molecular functions (MF), cellular components (CC), and gene-associated signaling pathways, GO and KEGG functional enrichment analyses and visualizations were carried out in the R environment using the R packages “clusterProfiler” and “ggplot2”.

### 2.7 Hub gene validation

#### 2.7.1 Establishment of NAFLD cell models

After being thawed, Alpha Mouse Liver 12 (AML12) cells were cultivated for 24 hours at 37 °C with 5% CO2. Experiments were conducted after 2–3 stable passages. Cells were separated into two groups at about 80% confluence: a control group and a NAFLD group (OA: PA = 2:1), and they were then cultivated for an additional 24 hours. The complete medium composition comprised 88% DMEM/F12 medium (GIBCO, 11320033), 10% fetal bovine serum (GIBCO, A5256701), 1% ITS liquid medium supplement (Sigma, I3146), 1% penicillin-streptomycin antibiotic, and 40 ng/mL dexamethasone. The NAFLD group was treated with 1 mmol/L free fatty acid (FFA) solution (PA: OA = 1:2) (25).

#### 2.7.2 Oil red O staining

A modified Oil Red O staining kit (Beyotime, C0158S) was used for staining after the NAFLD cell model was established. Briefly, cells were fixed with a 4% paraformaldehyde solution for ten minutes after the cell culture medium was discarded, and then they were washed twice with PBS. For 20 seconds, cover the cells with the appropriate volume of staining wash solution. After removing the staining wash solution, apply the proper amount of modified Oil Red O staining solution and let it sit for ten to twenty minutes. After removing the modified Oil Red O staining solution, add the proper amount of staining wash solution and let it stand for 30 seconds. Wash with PBS for 20 seconds after discarding the stained wash solution. Discard the wash solution, uniformly cover the cells with PBS, and observe and photograph under a microscope (Color Camera Nikon DS-Fi3).

#### 2.7.3 Establishment of a mouse model for NAFLD

Male C57BL/6 mice that were 6 to 8 weeks old and fed either a high-fat diet or a standard diet were used in this study. To create a mouse model of NAFLD, this regimen was followed for a total of eight weeks. The high-fat diet consisted of a commercial rodent chow containing 60 kcal% fat (Dyets, HF60). All animal experiments were approved by the Biomedical Research Ethics Subcommittee of Henan University.

#### 2.7.4 RT-qPCR validation

Total RNA was extracted from samples (cells or mouse liver tissue). The NanoPhotometer^®^ N60 was used to test the concentration of extracted RNA. Reverse transcription was then performed using the BIO-RAD T100™ Thermal Cycler, with product concentration measured by the NanoPhotometer^®^ N60. Enzyme-free ddH2O was used to dilute the reverse transcription product eight to ten times. The BIO-RAD CFX96™ Real-Time System device was then used to conduct PCR reactions. The single-peaked PCR amplification specificity was verified by melt curve analysis. Using GAPDH as the internal control standard, the obtained Ct values were examined using the 2^(-ΔΔCt) technique to determine relative mRNA expression levels. **Supplementary Table 1** contains primer sequences.

### 2.8 Statistical analysis

R software (version 4.4.3) and GraphPad Prism 10 were used for all statistical analyses and graphical displays. Based on the normality of the data, two statistical techniques were chosen: Wilcoxon tests for non-normally distributed data and t-tests for normally distributed data. In the training cohort, DEGs were identified based on the thresholds of an adjusted *P*-value < 0.05 and |log_2_FC| > 0.3. For functional enrichment, GO terms were considered significantly enriched with an adjusted *P*-value < 0.05, while KEGG pathways were identified using a threshold of an adjusted *P*-value < 0.1. In all other independent statistical comparisons, *P* < 0.05 was considered statistically significant.

## 3 Results

### 3.1 Identification of differentially expressed genes in NAFLD

**Figure 2:**
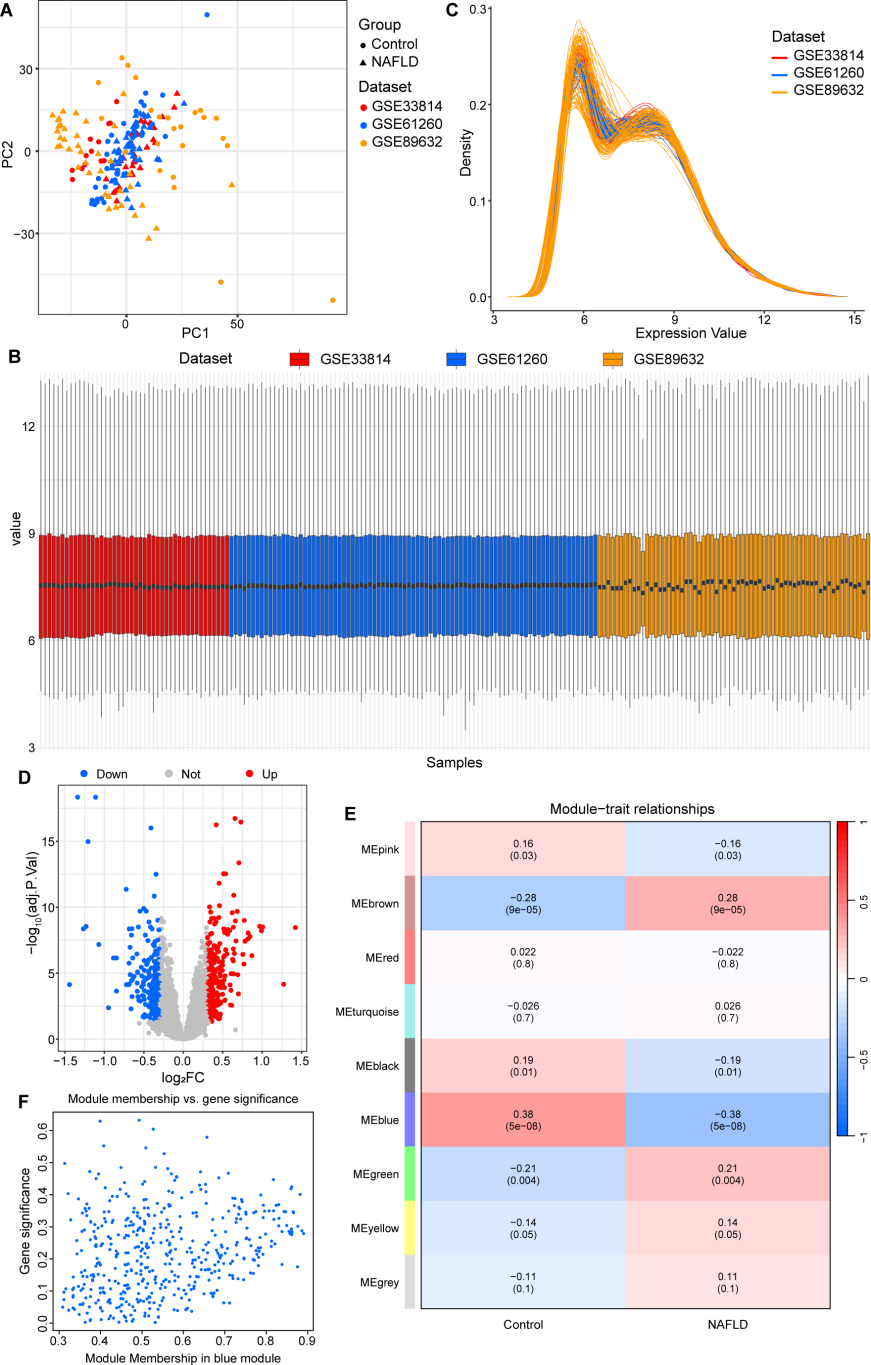
Batch correction analysis and differential expression analysis, along with the implementation of WGCNA and identification of key module genes. **(A)** PCA plot of the merged datasets after batch effect correction. **(B)** Boxplots of the three datasets after batch effect removal. **(C)** Density plot of expression values in the three datasets after batch correction. **(D)** Volcano plot of merged datasets. **(E)** Module-trait relationships: contrasting the NAFLD group with the control group. **(F)** The association between module membership and gene importance is seen in the scatterplot for the blue module. WGCNA, weighted Gene Co-expression Network Analysis. NAFLD, non-alcoholic fatty liver disease; PCA, Principal Component Analysis.

In order to address batch effects, we processed the datasets GSE33814, GSE61260, and GSE89632 using the “sva” R package and combined them into a new dataset. This new dataset comprised 75 healthy control cases and 117 NAFLD samples. The efficacy of the batch effect removal was validated through multiple quality control metrics: Principal Component Analysis (PCA) confirmed that samples no longer clustered by study origin, indicating successful elimination of technical variation; boxplot analysis demonstrated consistent median expression values and normalized distributions across all samples; and density distribution plots showed highly overlapping expression profiles across the three datasets. (**Figures 2A–C**; **Supplementary Figures 1A–C**) To explore DEGs between healthy controls and NAFLD cases, we effectively deployed the “limma” R package for this analysis. 451 genes with differential expression (|log_2_FC| > 0.3) were found, of which 250 were upregulated and 201 were downregulated (**Figure 2D**).

### 3.2 WGCNA implementation and key modular gene identification

WGCNA was applied to construct co-expression networks within the dataset, identifying modules closely associated with NAFLD. The built co-expression modules were reasonable, as shown by the unscaled R² reaching 0.9 when Soft was set to 6 (**Supplementary Figure 2A**). Using hierarchical clustering and optimal dynamic tree cutting techniques, several co-expression modules were found (**Supplementary Figure 2B**).

The associations between various gene co-expression modules (color-coded) and NAFLD are shown in the module–phenotype heatmap, where each cell represents the correlation coefficient and significance level between a particular module and the NAFLD phenotype (**Figure 2E**). The co-expression networks created for the NAFLD and control groups jointly identified nine gene co-expression clusters (**Figure 2E**). The blue module showed the strongest association with NAFLD among these (cor = - 0.38, *P* = 5e - 08). Module membership and gene significance inside the blue module showed a strong positive link in a scatter plot, with a correlation coefficient of 0.25 (*P* < 3.8e-08). This suggests that as a gene’s inclusion in the blue module rises–reflecting greater similarity between its expression pattern and the module’s characteristic expression pattern–its association with NAFLD and its potential biological significance for this trait also rise (**Figure 2F**). Ultimately, the blue module collectively identified 471 genes associated with NAFLD.

### 3.3 Acquisition and enrichment analysis of intersecting genes

**Figure 3:**
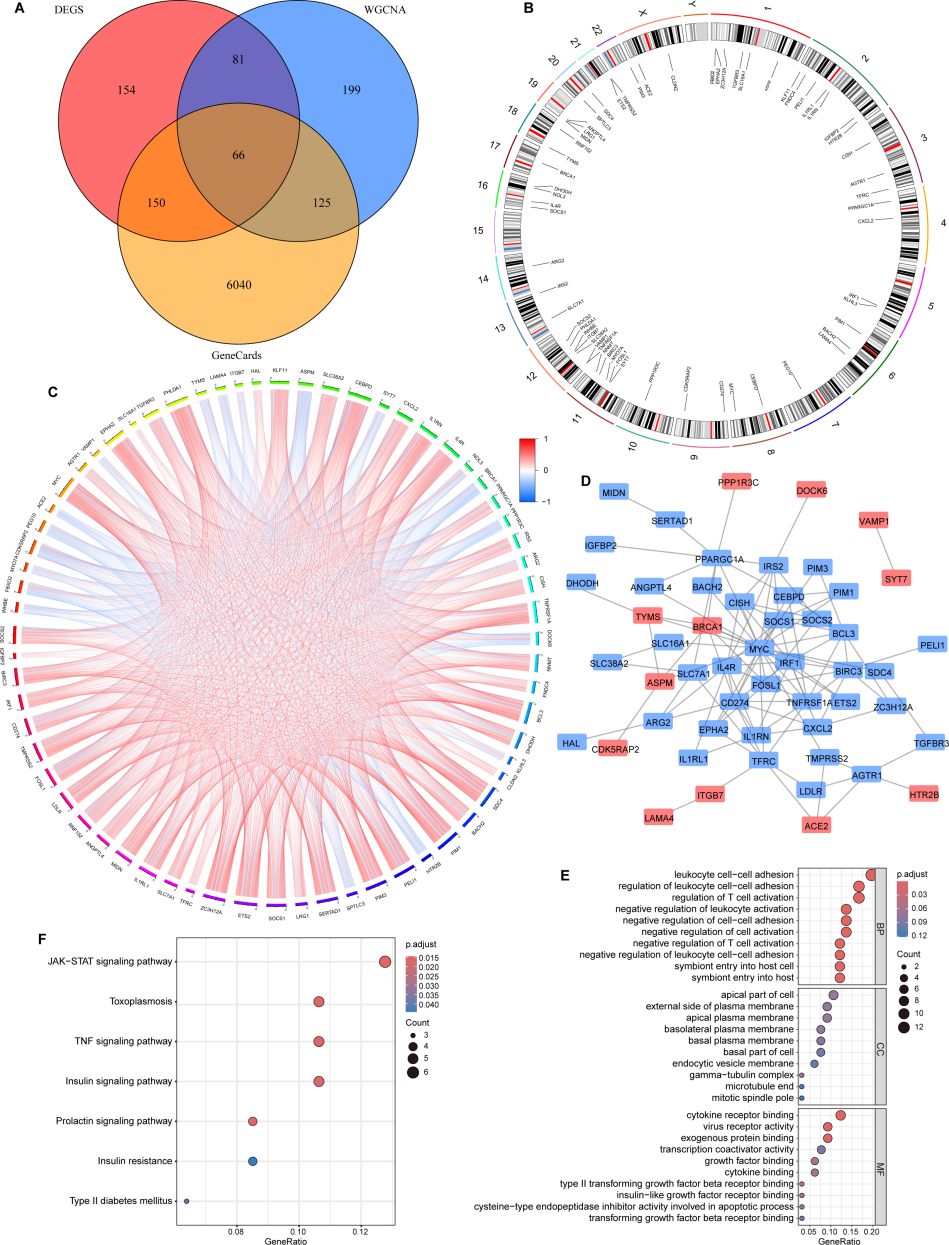
66 intersecting genes were acquired and their functional enrichment was examined. **(A)** 66 intersection genes of Genecards, WGCNA and DEGs. **(B)** Genosphere map of intersection genes. **(C)** The circle plot illustrates the correlations among the 66 intersecting genes. **(D)** The PPI network depicts the relationships among the intersecting genes. **(E)** GO enrichment analysis of 66 intersection genes. **(F)** KEGG enrichment analysis of 66 intersection genes. WGCNA, weighted Gene Co-expression Network Analysis. DEGs, differentially expressed genes.

The 451 DEGs, 6,381 genes associated with circulating insulin degradation, and 471 key module genes were intersected to produce 66 overlapping genes (**Figure 3A**). The chromosomal locations of each gene are depicted in the circular plot (**Figure 3B**). Intergenic correlations were calculated and visualized using the “corrplot” R package (**Figure 3C**), revealing coordinated expression patterns among the candidate genes. To investigate the functional crosstalk between these genes, a protein-protein interaction (PPI) network was constructed via the STRING database and visualized using Cytoscape (**Figure 3D**). Notably, genes such as ASPM, TYMS, and BRCA1 were significantly up-regulated (red nodes) within the network, whereas AGTR1, IL1RN, IGFBP2 and SOCS1, were down-regulated (blue nodes). GO enrichment analysis (**Figure 3E**) provided insights into the multi-dimensional roles of these genes. The BP terms were significantly enriched in leukocyte cell-cell adhesion and regulation of T cell activation, highlighting the inflammatory component often associated with metabolic dysfunction. MF analysis revealed enrichment in cytokine receptor binding and growth factor binding, which are critical for intra-hepatic signaling. Crucially, KEGG pathway enrichment analysis (**Figure 3F**) demonstrated that these intersecting genes are deeply involved in metabolic and inflammatory signaling pathways relevant to NAFLD. Specifically, significant enrichment was observed in the Insulin signaling pathway, TNF signaling pathway, and JAK-STAT signaling pathway. Furthermore, the analysis highlighted associations with Insulin resistance and Type II diabetes mellitus, suggesting that these genes may drive the progression of metabolic disorders through the impairment of insulin sensitivity and the activation of chronic inflammatory cascades.

To assess immune variations, we performed immune infiltration analysis (**Supplementary Figure 3A**). Compared with the control group, the NAFLD group showed significant alterations in several immune cell populations. In particular, Macrophages M1 were increased, whereas other immune cell subsets, including Neutrophils and Dendritic cells activated, also differed between groups. Correlation analysis of the intersecting genes with immune cell types revealed notable associations involving Macrophages M1, Neutrophils, and Dendritic cells activated (**Supplementary Figure 3B**). These findings offer computational estimates of immune cell dynamics and serve as a basis for generating biological hypotheses, which warrant further validation using high-resolution techniques such as liver-specific single-cell RNA sequencing.

### 3.4 Building and assessing machine learning models

**Figure 4:**
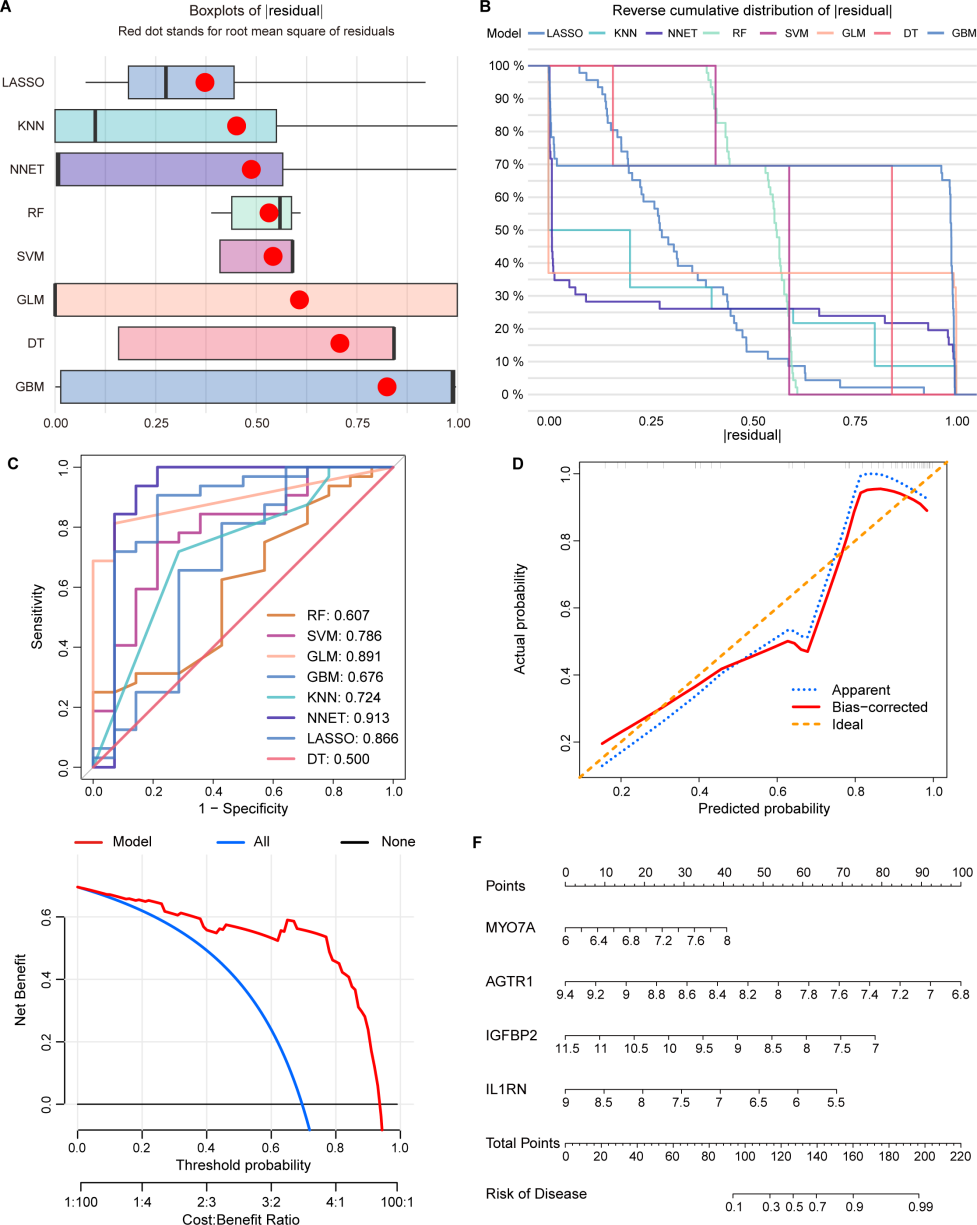
Construction and evaluation of eight machine models and Build a prediction model. **(A)** Each machine learning model’s residuals were displayed in boxplots. The root mean square error (RMSE) was shown by the red dot. **(B)** Each machine learning model’s cumulative residual distribution. **(C)** ROC curves of different models in the external test set. **(D)** Calibration curve of the optimal model in the external test set **(E)** Decision curve analysis of the optimal model in the external test set. **(F)** Using the 4-gene GLM model, a nomogram is constructed to predict the risk of NAFLD. NAFLD, non-alcoholic fatty liver disease.

Based on the candidate intersecting genes, we constructed eight machine learning prediction models (SVM, RF, GLM, GBM, KNN, NNET, LASSO, and DT). We implemented a Nested-CV framework, where feature selection and hyper-parameter tuning were strictly performed within the inner loop using the training cohort. Subsequently, the DALEX package was utilized to perform interpretative analyses on these models. Residual distribution plots were generated to evaluate their goodness-of-fit and stability. Results indicated that the GLM and LASSO models exhibited the most concentrated and stable residual distributions, with GLM showing superior fitting performance and lower overall residual magnitudes (**Figures 4A, B**). To assess the generalizability of the models, we conducted external validation strictly on an untouched independent dataset (GSE48452). The discriminative performance was evaluated using both ROC. As shown in the ROC curves (**Figure 4C**), the GLM model demonstrated robust diagnostic efficacy with an AUC of 0.891, while the NNET model achieved 0.913. Despite the slightly higher nominal AUC of NNET, the GLM was identified as the optimal diagnostic model for this study due to its exceptional stability in residual analysis and its superior statistical interpretability for clinical biomarkers.

Furthermore, we systematically evaluated the clinical utility of the GLM-based signature on the external set. The calibration curve (**Figure 4D**) revealed a high degree of consistency between the predicted risks and actual occurrences, with minimal deviation, confirming the model’s reliable calibration. Simultaneously, DCA demonstrated that the model provides a substantial clinical net benefit across a wide range of threshold probabilities (**Figure 4E**). Finally, based on the feature importance and regression coefficients within the GLM framework, we identified the top four hub genes—MYO7A, AGTR1, IGFBP2, and IL1RN—as the core diagnostic signature. A nomogram was subsequently constructed using these four variables to facilitate clinical decision-making (**Figure 4F**). The high ranking and stable performance of these genes across nested iterations underscore their significant contribution to NAFLD pathogenesis and their potential as robust diagnostic biomarkers.

### 3.5 The best machine learning model explanation

**Figure 5:**
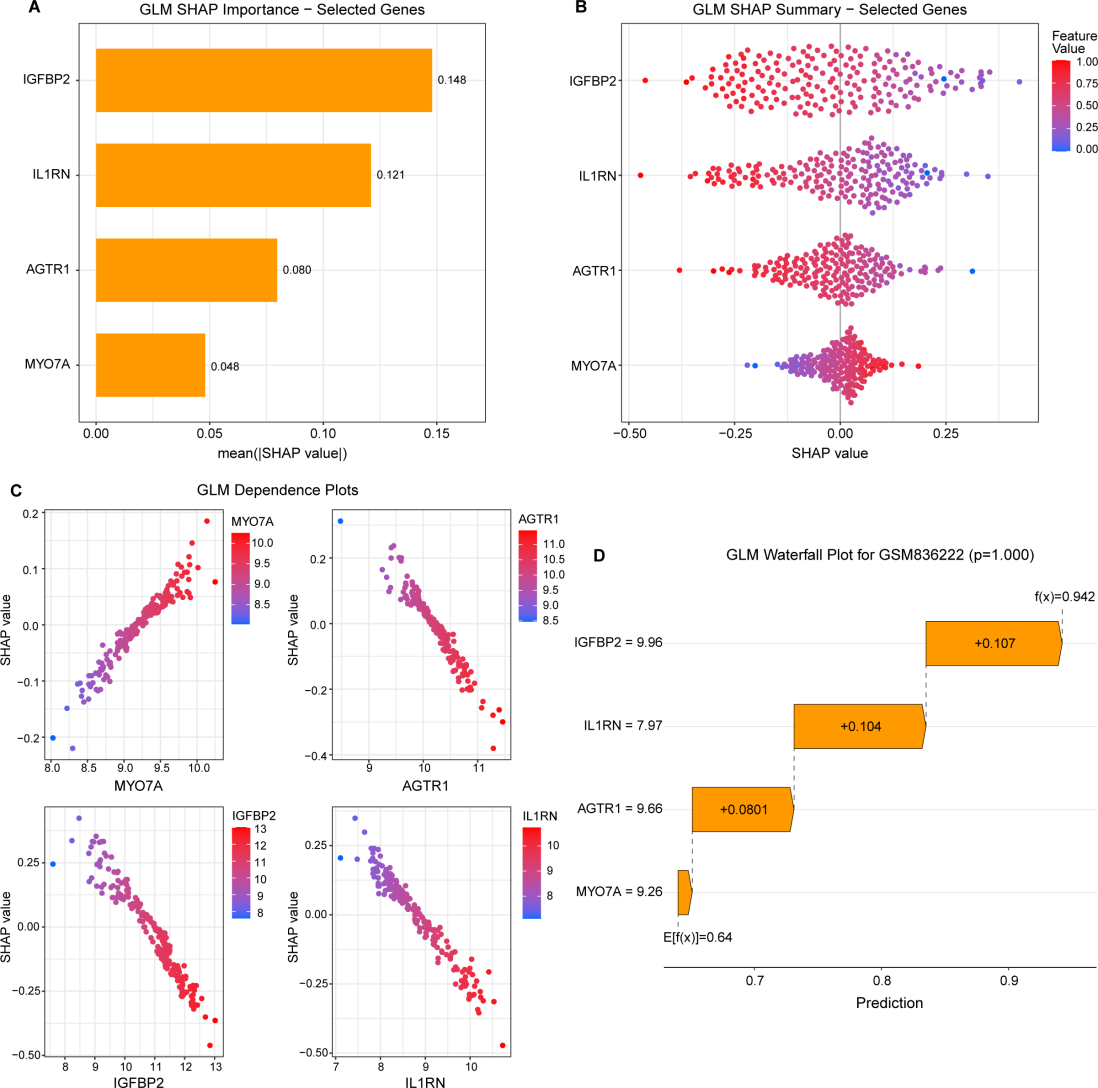
SHAP analysis of the best machine learning model. **(A)** SHAP bar plot showing each feature’s mean SHAP values. **(B)** Detailed SHAP value graphs that show how each attribute contributes to model predictions. **(C)** A SHAP bee plot that illustrates how features affect model output. **(D)** SHAP values for characteristics that point to a NAFLD prediction. SHAP, Shapley Additive Explanations. NAFLD, non-alcoholic fatty liver disease.

The SHAP method was employed to interpret the outputs of the optimal machine learning models by quantifying each variable’s contribution to the predictions. SHAP bar plots and summary (bee) plots (**Figures 5A, B**) indicate that within the GLM model, IGFBP2 exhibits the highest overall contribution (0.149), followed by IL1RN (0.121), AGTR1 (0.081), and MYO7A (0.048). The SHAP summary and dependence plots (**Figures 5B, C**) reveal the specific relationship between gene expression levels and predictive risk. While higher expression of MYO7A increases the SHAP value (positive contribution to the prediction), IGFBP2, IL1RN, and AGTR1 demonstrate an inverse relationship; specifically, lower expression levels of these three genes correspond to higher SHAP values, thereby increasing the probability of the target outcome. Individual Prediction Interpretation (**Figure 5D**) The contribution of each feature to the model’s predictive output for a representative sample (GSM836222) is illustrated in the waterfall plot (**Figure 5D**).

### 3.6 Hub genes validation and diagnostic efficacy in NAFLD

**Figure 6:**
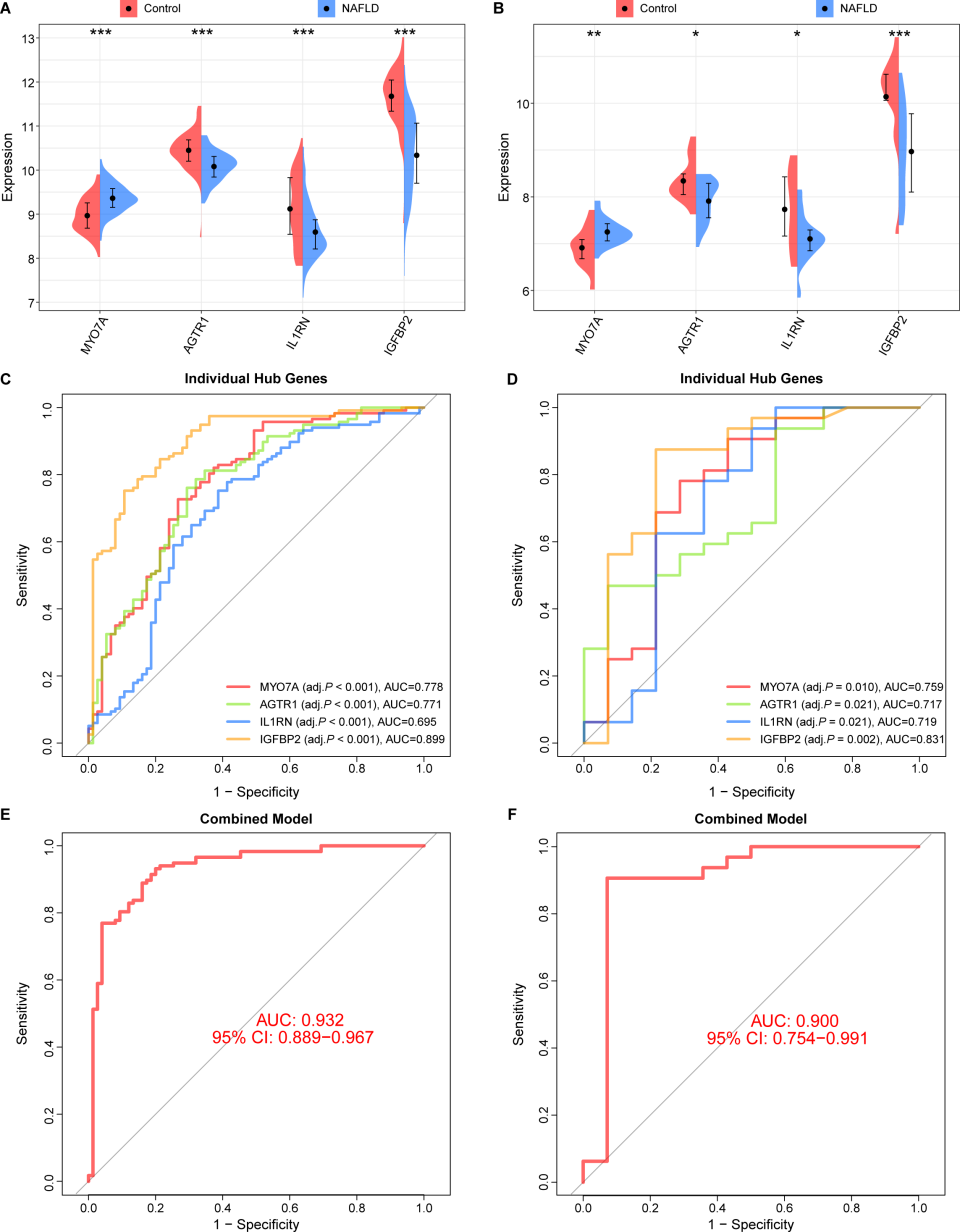
Hub genes validation and diagnostic effectiveness in NAFLD. **(A)** The expression of 4 hub genes in the training cohort. **(C, E)** ROC curves for individual pivotal genes and the diagnostic model within the training cohort. **(B)** The expression of 4 hub genes in the validation cohort. **(D, F)** ROC curves of 4 hub genes and diagnostic model in the validation cohort. (*P < 0.05, **P < 0.01, ***P < 0.001). NAFLD, non-alcoholic fatty liver disease.

We evaluated the expression levels of four identified hub genes—MYO7A, AGTR1, IL1RN, and IGFBP2—in both the training and validation cohorts. In the training cohort, MYO7A was significantly upregulated in the NAFLD group compared to healthy controls (*P* < 0.001), while AGTR1, IL1RN, and IGFBP2 were significantly downregulated (*P* < 0.001; **Figure 6A**). These expression patterns were consistently observed in the validation cohort, further confirming the robustness of these hub genes (**Figure 6B**). To assess their clinical utility, ROC curve analysis was performed. In the training cohort, IGFBP2 demonstrated the highest diagnostic accuracy with an AUC of 0.899, followed by MYO7A (AUC = 0.778) and AGTR1 (AUC = 0.771), while IL1RN yielded an AUC of 0.695 (**Figure 6C**). Similar diagnostic performance was maintained in the validation cohort, where all four genes exhibited AUC values exceeding 0.70 (**Figure 6D**). Notably, the diagnostic model integrating these four genes showed superior discriminative power, achieving AUC values of 0.932 (95% CI: 0.889–0.967) and 0.900 (95% CI: 0.754–0.991) in the training and validation cohorts, respectively (**Figures 6E, F**).

Furthermore, we explored the correlation between these hub genes and the immune microenvironment (**Supplementary Figure 4**). IL1RN exhibited the strongest correlations with multiple immune cell types, including significant positive correlations with activated mast cells and neutrophils, and a strong negative correlation with resting mast cells and CD8+ T cells (*P* < 0.001). Additionally, AGTR1 showed a significant positive correlation with neutrophils, while MYO7A and IGFBP2 displayed specific associations with activated dendritic cells and activated mast cells, respectively.

### 3.7 Cell and animal experiments

**Figure 7:**
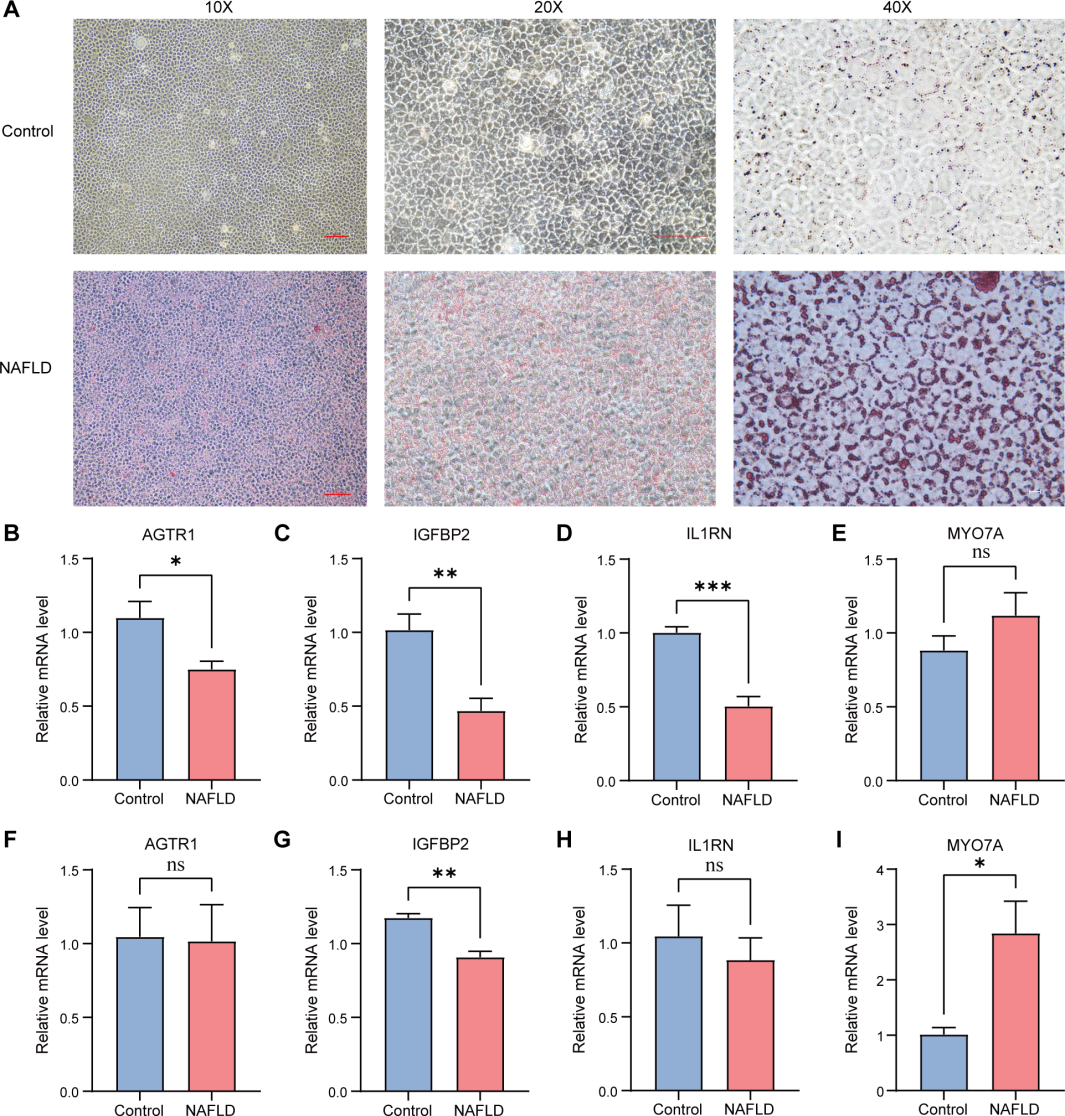
Cell (n=3) and animal (n=4) experiments **(A)** AML12 cells stained with Oil Red O under 10×, 20× and 40× magnification. **(B)** AGTR1 relative mRNA levels in NAFLD and control cells. **(C)** IGFBP2 relative mRNA levels in NAFLD and control cells. **(D)** IL1RN relative mRNA levels in NAFLD and control cells. **(E)** MYO7A relative mRNA levels in NAFLD and control cells. **(F)** AGTR1 relative mRNA levels in NAFLD and control mice. **(G)** IGFBP2 relative mRNA levels in NAFLD and control mice. **(H)** IL1RN relative mRNA levels in NAFLD and control mice. **(I)** MYO7A relative mRNA levels in NAFLD and control mice. NAFLD, non-alcoholic fatty liver disease. (*P < 0.05, **P < 0.01, ***P < 0.001).

To verify the predictive ability of hub genes, we performed RT-qPCR experiments using the AML12 cell line successfully established from the NAFLD model. As seen in **Figure 7A**, abundant orange-red lipid droplets were visible in NAFLD group cells, whereas only minimal background staining was observed in the control group. In line with the predicted trends seen in the previous gene expression study, RT-qPCR analysis showed significant differences in the expression levels of the AGTR1(95%CI: -0.692 – -0.006, *P* < 0.05), IGFBP2(95%CI: -0.885 –-0.212, *P* < 0.01) and IL1RN(95%CI: -0.690 – -0.311, *P* < 0.001) genes between the control and NAFLD groups (**Figures 7B–D**). Furthermore, the expression changes in MYO7A(95%CI: -0.276–0.748, *P* = 0.27) also corresponded with the previously predicted trends (**Figure 7D**). The expression trends of AGTR1(95%CI: -0.809 – 0.748, *P* = 0.93), IGFBP2(95%CI: -0.403 – -0.132, *P* < 0.01), IL1RN(95%CI: -0.878 – 0.554, *P* = 0.56), and MYO7A(95%CI: 0.181 – 3.473, *P* < 0.05) in the animal model were in line with the findings of the cellular experiment (**Figures 7F, G, H, I**), with IGFBP2 and MYO7A showing particularly significant expression differences.

## 4 Discussion

At present, affecting almost 25% of the world’s population, NAFLD is one of the most common chronic liver diseases worldwide, and its prevalence is still rising (15). Equally critically, hepatocellular carcinoma, cirrhosis, and liver fibrosis can develop from NAFLD. Public health systems and socioeconomic resources are heavily burdened by these severe hepatic complications, which significantly raise patients’ risk of liver-related and all-cause mortality (26, 27). Although the ‘multiple hits’ theory centered on lipid metabolism disorders, insulin resistance (IR), and chronic inflammatory responses has long been a research focus, the rapid advancement of multi-omics technologies in recent years—such as transcriptomics, genomics, and systems biological analysis—has further revealed the intricate and sophisticated molecular regulatory networks underlying NAFLD development.

We used gene sets linked to circulating insulin degradation from the GeneCards database, differentially expressed genes from three distinct cohorts in the GEO database, and important co-expression module genes found using WGCNA analysis in this study. Through the intersection of these three datasets, we were able to identify a group of potentially important genes that are probably involved in the pathophysiology and progression of NAFLD, offering fresh perspectives on the molecular mechanisms of NAFLD at the systems level. KEGG enrichment analysis of the 66 intersecting genes revealed their predominant enrichment in pathways closely linked to energy and lipid metabolism, including Insulin signaling pathway, TNF signaling pathway, and JAK-STAT signaling pathway. This suggests their potential functional roles in metabolic and inflammatory signaling pathways relevant to NAFLD. Previous studies indicated that hepatic oxidative stress and pro-inflammatory signaling cascades are key drivers of NAFLD formation and progression (28), and are closely associated with multiple immune cell populations, including macrophage/Kupffer-cell-related inflammatory responses, NK cells, dendritic cells, and neutrophils (29). In the present study, immune infiltration analysis also suggested alterations in several immune cell subsets in NAFLD, supporting the notion that the immune-inflammatory microenvironment is involved in NAFLD pathogenesis.

Another core aspect in the pathogenesis of NAFLD is the interaction between insulin metabolism abnormalities and hepatic lipid deposition (30). As a critical metabolic hormone, insulin homeostasis relies heavily on the liver’s capacity to uptake and degrade circulating insulin. When hepatic insulin degradation capacity is impaired, persistently elevated circulating insulin levels may ensue, thereby exacerbating insulin resistance, promoting lipogenesis and hepatic lipid accumulation, and establishing a vicious cycle of metabolic imbalance and fatty liver progression (31–33). Against this pathophysiological backdrop, we integrated circulating insulin degradation-related genes with NAFLD-associated differentially expressed genes, combining these with WGCNA screening results to identify pivotal genes mediating the link between NAFLD and insulin metabolism abnormalities. Building on this, we developed eight machine learning models to comprehensively model and screen features in the training cohort of the combined dataset: SVM, RF, GLM, GBM, KNN, NNET, LASSO, and DT. By comparing residual distributions and ROC curve performance across models in the training cohort, we identified GLM as superior in both model stability and discriminative efficacy, establishing it as the optimal diagnostic model. Further feature importance scoring within GLM yielded MYO7A, AGTR1, IGFBP2, and IL1RN as the four highest-scoring genes, designated as hub genes. Subsequently constructed nomograms, combined with calibration curves and DCA results, demonstrated that this model exhibits good discriminatory power, precise calibration, and considerable clinical net benefit in predicting NAFLD occurrence, proving its high potential for clinical application. Using the SHAP approach, we also determined each hub gene’s contribution to the machine learning prediction model. The genes were ranked in descending order of contribution as follows: IGFBP2, IL1RN, AGTR1, and MYO7A.

To validate the expression profiles of the bioinformatically identified hub genes, we employed both *in vitro* (cellular) and *in vivo* (animal) models of NAFLD. Morphological analysis via Oil Red O staining (**Figure 7A**) confirmed significant intracellular lipid accumulation in the NAFLD group compared to the control, demonstrating the successful establishment of the hepatic steatosis models.

IGFBP2 is recognized as a protective metabolic factor, with its levels inversely correlated with obesity, insulin resistance, and the severity of NAFLD (34). Recent studies have demonstrated that IGFBP2 functions as an endogenous protector against hepatic steatosis; its deficiency exacerbates lipid accumulation by activating the EGFR-STAT3 signaling pathway, which in turn promotes the expression of lipogenic genes like Srebf1 (35). Additionally, other members of the IGFBP family have been shown to attenuate steatosis through the AMPK pathway, a master regulator of energy homeostasis (36). In the present study, mRNA levels of IGFBP2 were significantly downregulated in both cellular (**Figure 7C**) and animal models (**Figure 7G**) (*P* < 0.01). These findings are concordant with existing literature, suggesting that the loss of IGFBP2 impairs the liver’s metabolic defenses, serving as a critical driver of NAFLD progression.

IL1RN is a naturally occurring anti-inflammatory cytokine that competitively binds to the IL-1 receptor, thereby neutralizing the pro-inflammatory activities of IL-1β (37). The transition from simple steatosis to NAFLD is largely governed by uncontrolled inflammatory responses; notably, experimental studies have shown that IL1RN deficiency leads to accelerated development of hepatic inflammation and steatosis (38). Our results indicated a significant reduction in IL1RN expression in the cellular model (**Figure 7D**, *P* < 0.001), with a consistent downward trend observed in the animal model (**Figure 7H**). This suppression suggests that diminished IL1RN levels may increase the inflammatory susceptibility of hepatocytes during the early stages of NAFLD.

As a major effector receptor of the renin-angiotensin system (RAS), AGTR1 has been widely implicated in promoting hepatic oxidative stress and fibrogenic signaling. Experimental studies in NASH models have shown that pharmacological blockade of AGTR1 attenuates oxidative stress, hepatic stellate cell activation, and liver fibrosis, supporting its pathogenic role in liver injury (39, 40). In our study, AGTR1 mRNA levels were significantly decreased in the cellular model (**Figure 7B**, *P* < 0.05), while remaining relatively stable in the animal model (**Figure 7F**). This pattern may indicate a context-dependent adaptive response in hepatocytes under acute lipid loading, possibly serving to limit excessive RAS-mediated signaling and cellular injury.

MYO7A is an unconventional actin-based motor protein implicated in intracellular cargo transport and organelle positioning, with established roles in the movement of melanosomes and phagosomes in retinal pigment epithelial cells (41, 42). Given that lipid droplet remodeling is a central feature of NAFLD progression (43), alterations in motor- and trafficking-related genes may contribute to disturbed hepatic lipid handling and tissue reorganization during disease development (44). In our study, MYO7A expression remained unchanged in the cellular model (**Figure 7E**), whereas it was significantly upregulated in the NAFLD mouse model (**Figure 7I**, *P* < 0.05). However, because direct evidence linking MYO7A to hepatic lipid droplet dynamics is still limited, this interpretation should be regarded as hypothesis-generating rather than conclusive.

In addition to their biomarker potential, the identified hub genes may also provide clues for therapeutic development. Specifically, the observed changes in IGFBP2, IL1RN, and AGTR1 suggest that restoration of metabolic protection, attenuation of IL-1-driven inflammation, and inhibition of RAS-related signaling may represent potential therapeutic directions in NAFLD. By contrast, the role of MYO7A remains less well defined and should currently be regarded as hypothesis-generating rather than directly targetable. Overall, our findings highlight several disease-relevant pathways that may be prioritized for future therapeutic exploration, although further mechanistic and translational studies are required before clinical application can be considered.

In conclusion, this study used systems biology analysis and machine learning techniques to identify four hub genes associated with circulating insulin degradation in NAFLD. These genes should be considered candidate biomarkers and potential therapeutic targets rather than definitively validated clinical markers, and they provide a useful basis for future mechanistic and translational studies. However, our research also presents several limitations that cannot be overlooked. First, all data used in this study were obtained from the GEO public database. Although these datasets were extensive and well curated, their reliance on processed public transcriptomic data rather than prospectively collected clinical samples may introduce selection bias and limit the generalizability of the findings. Second, although our cellular and animal models showed trends broadly consistent with the bioinformatics analyses, some results did not reach statistical significance, which may be attributable to limited sample sizes and underscores the need for further validation in larger, well-characterized cohorts, particularly human clinical samples. Furthermore, although this study provides preliminary and hypothesis-generating evidence regarding the potential roles of these hub genes in NAFLD, additional *in vivo* and *in vitro* functional experiments are still required to clarify their precise roles in the etiology and progression of the disease. Moreover, the findings from the immune infiltration analysis require further confirmation using liver-specific single-cell datasets or dedicated validation studies.

## Data Availability

Publicly available datasets were analyzed in this study. This data can be found here: https://www.ncbi.nlm.nih.gov/geo/.

## Glossary

NAFLD: non-alcoholic fatty liver disease
WGCNA: weighted gene co-expression network analysis
MASLD: metabolic dysfunction-associated steatotic liver disease
MAFLD: metabolic dysfunction-associated fatty liver disease
DEGs: differentially expressed genes
GEO: Gene Expression Omnibus
RF: Random Forest
SVM: Support Vector Machine
GLM: Generalised Linear Model
GBM: Gradient Boosting Machine
KNN: K-Nearest Neighbours
NNET: Neural Network
LASSO: Least Absolute Shrinkage and Selection Operator
DT: Decision Tree
Nested-CV: nested cross-validation
AUC: area under the curve
DCA: decision curve analysis
TPR: true positive rate
FPR: false positive rate
SHAP: SHapley Additive explanation
GO: Gene ontology
KEGG: Kyoto encyclopedia of genes genomes
BP: biological processes
MF: molecular functions
CC: cellular components
AML12: Alpha Mouse Liver 12
PCA: Principal Component Analysis
