# Supplementary material for: Identification of candidate biomarkers for NAFLD through bioinformatics analysis and machine learning based on circulating insulin degradation-associated genes
Source: Front Endocrinol (Lausanne). 2026 May 12;17:1774997. doi: 10.3389/fendo.2026.1774997 (PMC13201111; doi:10.3389/fendo.2026.1774997)
Supplement: Supplementary file 1 [file DataSheet1.docx]

**1 Supplementary Figures** **and Tables**

## Supplementary Figures


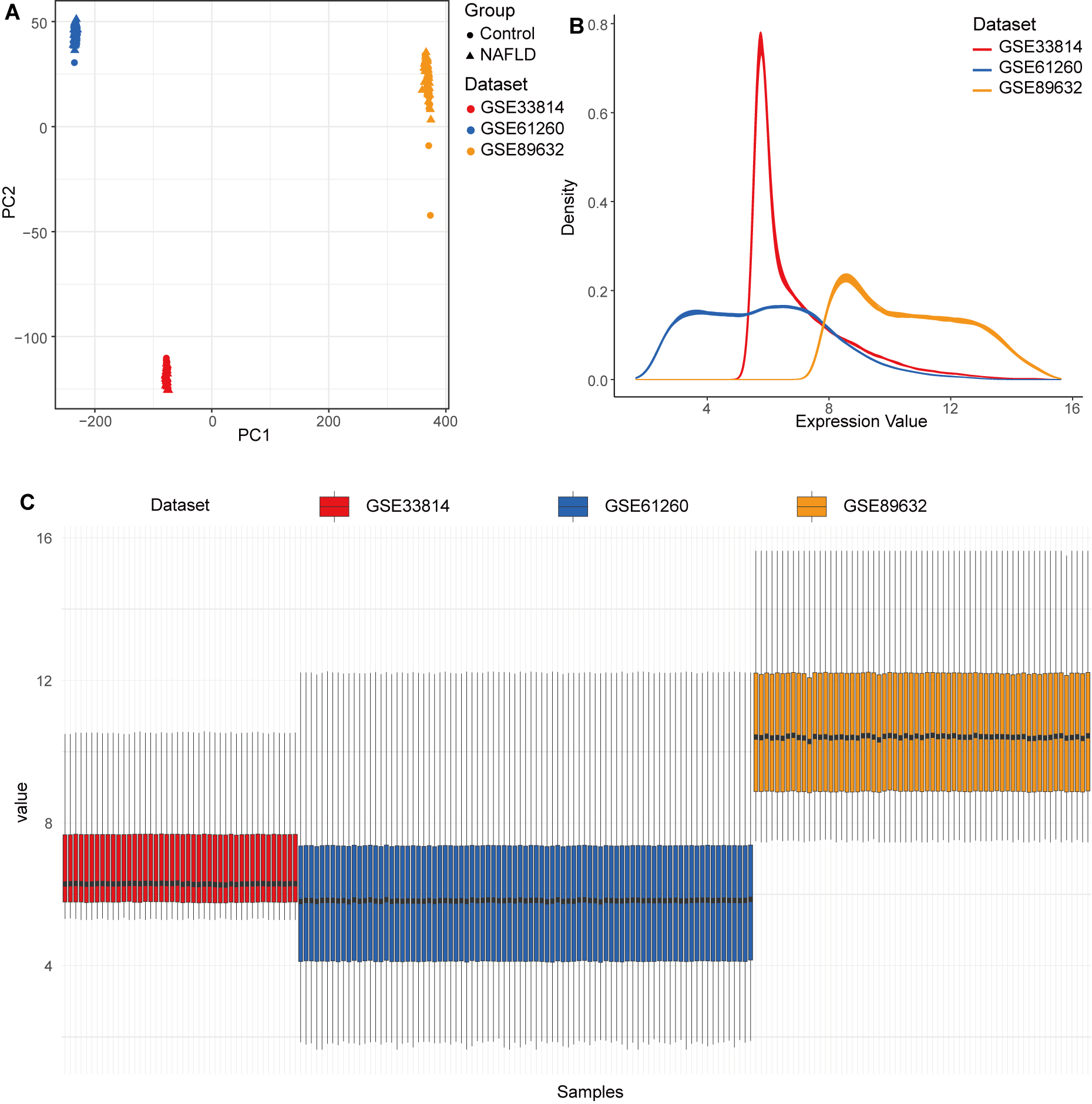


Supplementary Figure 1. Distribution of raw data before batch effect correction. (A) PCA plot: Samples clustered by dataset (GSE33814, GSE61260, GSE89632), indicating significant batch effects. (B) Density plot: Distribution of gene expression levels across the three datasets. (C) Boxplot: Comparison of expression value ranges and medians among samples.


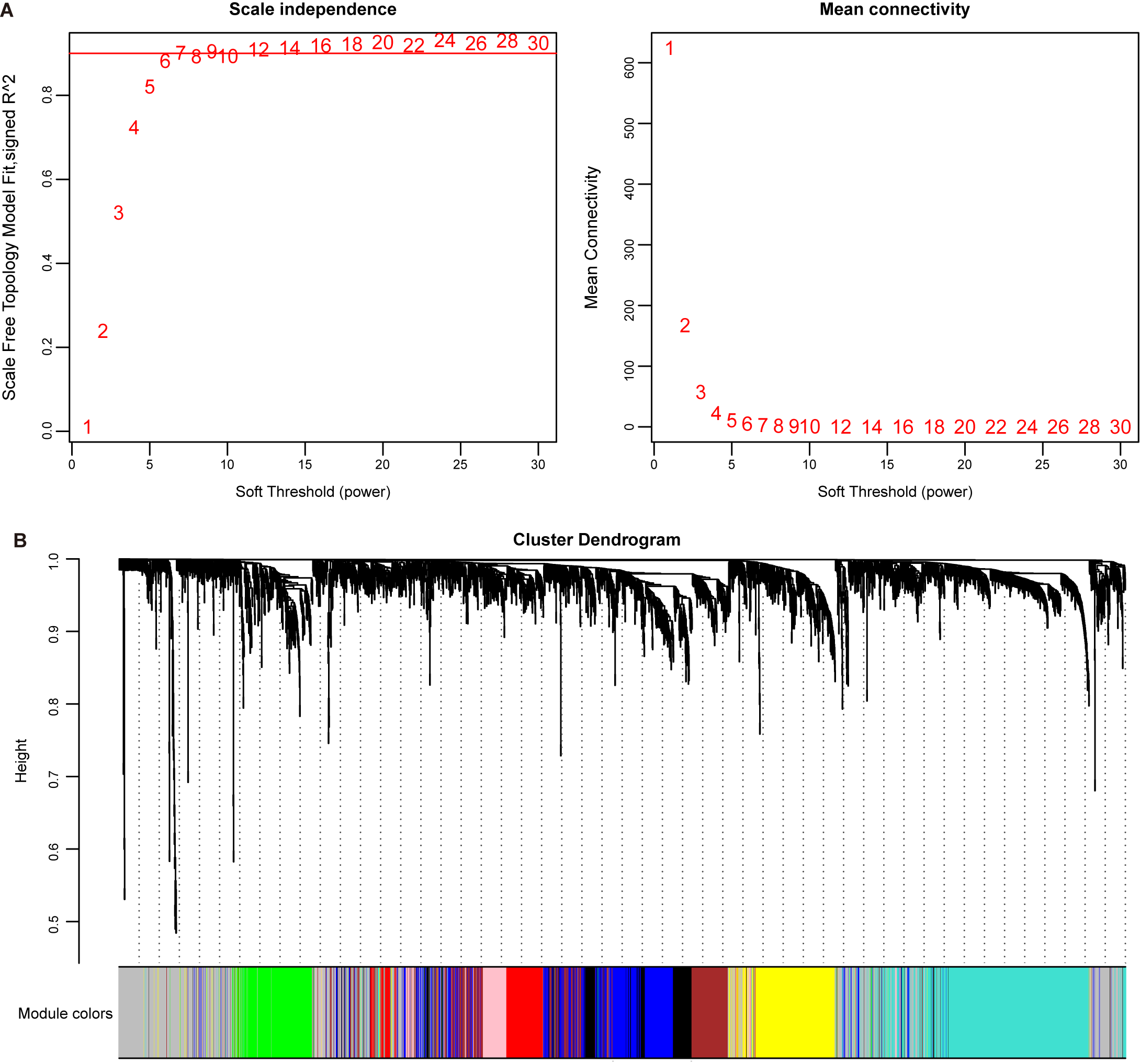


Supplementary Figure 2. Screening of gene modules (A) Analysis of network topologies for different soft-thresholding powers based on the scale-free fit index and mean connectivity. (B) Cluster dendrogram of co-expression modules.


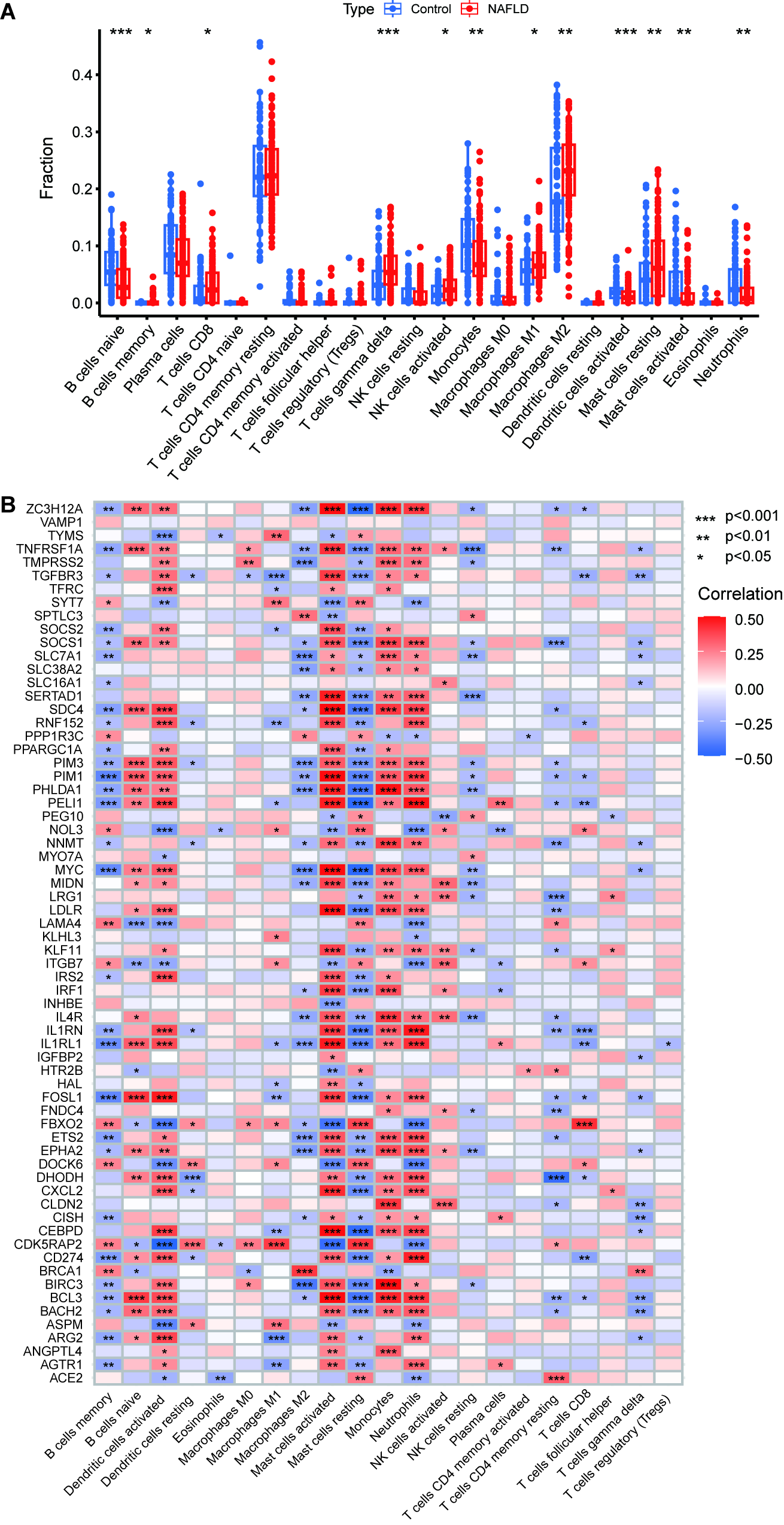


Supplementary Figure 3. Immune infiltration analysis in NAFLD. (A) Boxplots showing differences in immune infiltration between NAFLD and Control. (B) Correlation analysis between the 66 intersecting genes and immune cells. NAFLD, non-alcoholic fatty liver disease. (*p< 0.05, **< 0.01, ***p< 0.001, ****p< 0.0001)


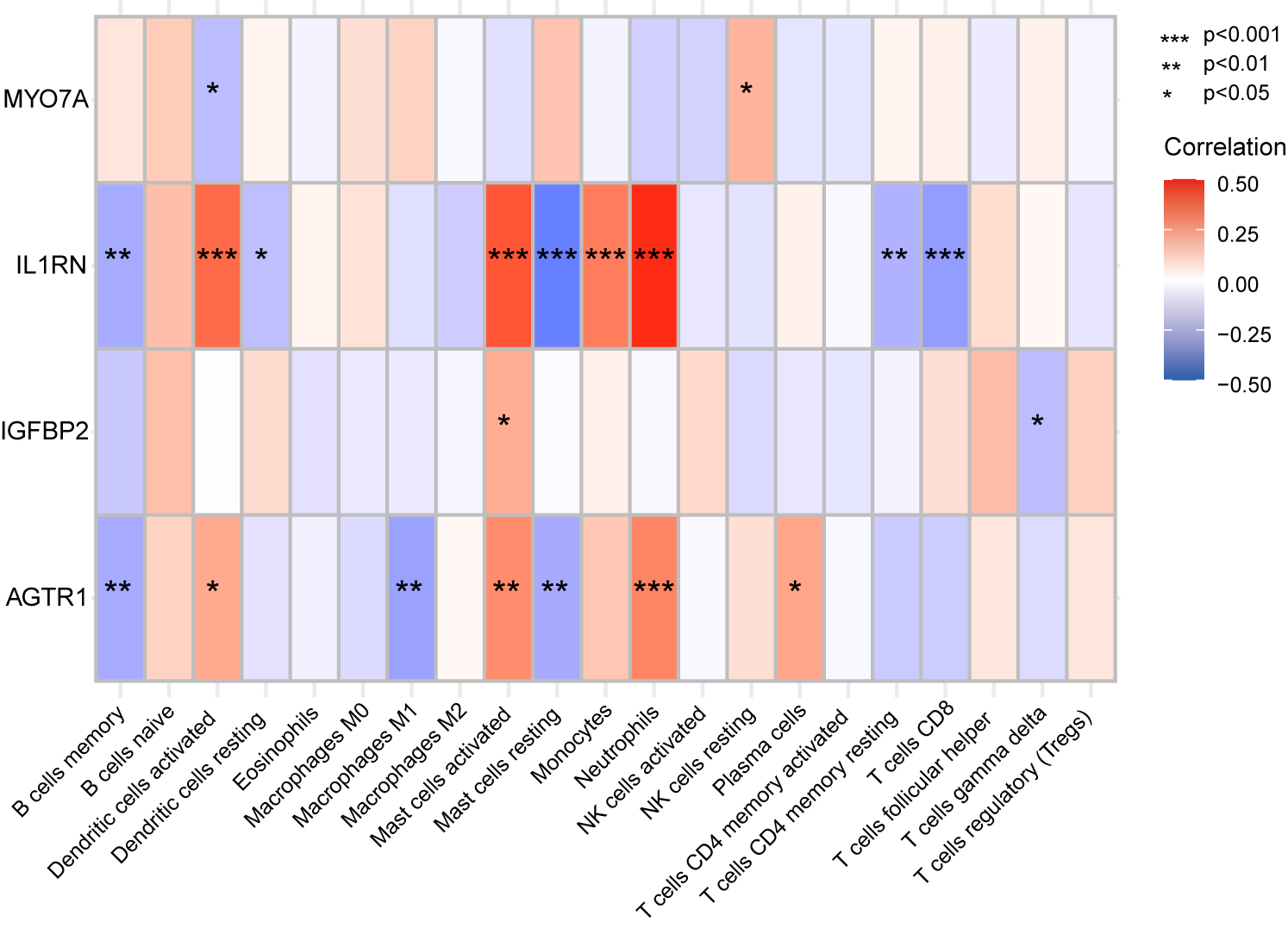


Supplementary Figure 4 Correlation analysis between the hub genes and immune cells.

## Supplementary Table

Supplementary table 1 The primer sequences for RT-PCR

|  | Forward | Reverse |
| --- | --- | --- |
| MYO7A | GCTTCAAAGACAGGTCCAACTTC | AGCCAAGACGAATCAGCTCATAG |
| AGTR1 | TGCTCTCCCGGACTTAACATATG | TCTCTTCTGGTGATGGCTTTCTT |
| IGFBP2 | AACATCTCTACTCCCTGCACATC | TCTCCTGCTGCTCGTTGTAGAA |
| IL1RN | CAAGATGCAAGCCTTCAGAATCT | GGATGCCCAAGAACACACTATGA |
| GAPDH | CATCACTGCCACCCAGAAGACTG | ATGCCAGTGAGCTTCCCGTTCAG |
